# Cyclic Ion Mobility Mass Spectrometry Distinguishes Anomers and Open-Ring Forms of Pentasaccharides

**DOI:** 10.1007/s13361-019-02168-9

**Published:** 2019-04-11

**Authors:** Jakub Ujma, David Ropartz, Kevin Giles, Keith Richardson, David Langridge, Jason Wildgoose, Martin Green, Steven Pringle

**Affiliations:** 1 Waters Corporation, Stamford Avenue, Altrincham Road, Wilmslow, SK9 4AX UK; 2 INRA, UR1268 Biopolymers Interactions Assemblies, Rue de la Géraudière, B.P. 71627, F-44316 Nantes, France

**Keywords:** Ion mobility, Mass spectrometry, Saccharides, Fragmentation, Anomers

## Abstract

There is increasing biopharmaceutical interest in oligosaccharides and glycosylation. A key requirement for these sample types is the ability to characterize the chain length, branching, type of monomers, and importantly stereochemistry and anomeric configuration. Herein, we showcase the multi-function capability of a cyclic ion mobility (cIM) separator embedded in a quadrupole/time-of-flight mass spectrometer (Q-ToF MS). The instrument design enables selective activation of mobility-separated precursors followed by cIM separation of product ions, an approach analogous to MS^n^. Using high cIM resolution, we demonstrate the separation of three isomeric pentasaccharides and, moreover, that three components are present for each compound. We show that structural differences between product ions reflect the precursor differences in some cases but not others. These findings are corroborated by a heavy oxygen labelling approach. Using this methodology, the identity of fragment ions may be assigned. This enables us to postulate that the two main components observed for each pentasaccharide are anomeric forms. The remaining low abundance component is assigned as an open-ring form.

**Electronic supplementary material:**

The online version of this article (10.1007/s13361-019-02168-9) contains supplementary material, which is available to authorized users.

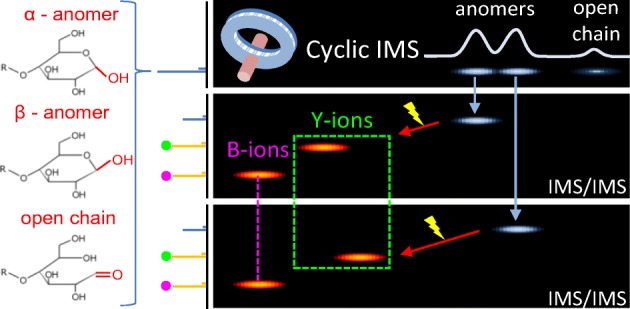

## Introduction

Carbohydrates are essential to life on earth. These biomolecules are found in every phylogenetic group. Foremost, they are components of most hybrid biomolecules. Associated with proteins, proteoglycans are present at the cell–extracellular interfaces of virtually all animals. They regulate many biological functions, including organogenesis and growth control, cell adhesion, signaling, inflammation, tumorigenesis, and interactions with pathogens [1]. Associated with lipids, the lipopolysaccharides are involved in cell signaling and infection mechanisms of Gram-negative bacterial as well as their structural integrity [2]. Furthermore, as pure carbohydrate structures, polysaccharides are the major components of all terrestrial and marine plants. They have essential biological roles as major components of the primary and secondary walls that surround plant cells. Here, polysaccharides ensure the maintenance of the structural rigidity of plants [3] as well as being involved in defense and signaling mechanisms [4]. All the aforementioned classes of carbohydrates are subject to intensive studies due to interest in their biological functions and because they constitute a huge reservoir of molecules of high structural variety [5]. They are extremely valuable in many industries such as the food industry and also in the value-added sectors such as pharmaceuticals and cosmetics [6]. As with other classes of compound, determination of structure is crucial for understanding their biological function and possible uses in industrial applications. Particularly relevant are the variations of structure within organs, subcellular structures, and between different stages of plant development. In addition to the degree of polymerization (DP), other chemical and structural features of polysaccharides are key elements for their function and end-use. These include the nature of the osidic monomers, the linkage between the building blocks, and the possible modifications by chemical substituents. However, the structural characterization of polysaccharides remains a challenge for analytical chemistry due to the complexity and heterogeneity of the structures.

NMR has been the technique of choice for analyzing carbohydrates for several decades [7]. However, this approach has several limitations mainly related to the sensitivity, small sample volumes, high polydispersity, and polymolecularity of the carbohydrates. Some of these problems are alleviated by mass spectrometry (MS) approaches. MS-based methods have long been established in the analysis of carbohydrates and provide key advantages to address the structural elucidation of polysaccharides with remarkable sensitivity, high information content, and high tolerance to mixtures when coupled with liquid chromatography techniques [8–11]. Nevertheless, determination of the anomeric configuration of the linkage between subunits remains a major challenge. The glycosidic bond can exhibit α- and β-anomeric configurations that strongly impacts on the properties of the carbohydrate. The identical mass of α- and β-linked species renders them indistinguishable by mass spectrometric analysis. Several gas phase methods have been combined with MS in order to differentiate between isomeric compounds. Particularly attractive are action spectroscopy approaches in the ultra-violet (UV) and infra-red (IR) regions. Here, the vibrational [12] and electronic [13] properties of mass-selected ions can be probed in detail. In their recent study, Schindler et al. have utilized vibrational spectroscopy to show that the stereochemistry of the glycosidic bond is retained in the product ions, following dissociation [14]. A related technique of UV photo-dissociation (UVPD) coupled with MS has been shown to yield diagnostic product ions and facilitate structural assignments [15]. Ion mobility spectrometry (IMS) coupled with MS detection (IM-MS) is another attractive approach in deciphering polysaccharide structures. Structural isomers, often encountered in the case of carbohydrates, can exhibit different mobility [16–18]. The latter is valid for both the precursor and the product ions, following dissociation. Notably, Hofmann et al. shown that IM-MS can be used to define the anomerism of disaccharides, after a derivatization, based on the IM measurement of the compounds [19]. Gray et al. have utilized IM separation and MS/MS to show that the anomeric configuration of disaccharides is preserved after collision-induced dissociation (CID), a finding corroborated further by IR spectroscopy experiments [20]. The utility of IM technology has been expanded further via a tandem IM approach, parallel to MS/MS analysis. A tandem IM instrument, developed by Li et al., was used to study a mixture of pentasaccharides and revealed the isomeric heterogeneity of the carbohydrate product ions [21]. Gaye et al. utilized a tandem IM method to investigate the collision cross sections (CCSs) of the isomeric product ions of isomeric precursor species (mono-, di-, and tri-saccharides); the product ion data were used to differentiate between precursors [22]. The use of a high-resolution IM-MS platform for the analysis of mixtures of saccharides has been demonstrated by Deng et al. [23]. Interestingly, multiple components of pentasaccharide species were resolved. Recently, we explored the use of high-resolution multi-pass cyclic IM (cIM) instrumentation [24] for analysis of the same saccharide mixture [25]. These analyses revealed multiple components for each pentasaccharide, in line with those reported by Deng et al. In addition, we performed IMS/IMS experiments on the two mobility-separated peaks of the maltopentaose. Remarkably, the ATDs of some of the product ions indicated the structural difference of the precursors had been retained whereas others did not. This was a clue that those product ions exhibiting the difference had retained the reducing end of the saccharide and thus contained anomeric information—a structural difference responsible for the separation of precursors [25]. A further study by Nagy et al. showed that carbohydrates containing the reducing end can exhibit two (or more) features in IM spectra, while non-reducing carbohydrates presented as a single peak [26]. The above studies hint that high-resolution IMS could resolve the anomeric forms of sodiated carbohydrates; however, sodium coordination itself could play a major role in this phenomenon. Understandably, metal cation coordination may vary with anomeric configuration, which could amplify the mobility difference of anomers. Conversely, different coordination sites *themselves* could account for the multiple “features” observed in the IM spectra. In this report, we attempt to explore the origin of this effect. Using a heavy oxygen labelling strategy [27], we are able to identify product ions containing the reducing end of the pentasaccharides. Using the high-resolution IM separation capability, we resolve two main components and also a third, low-intensity species. With IMS/IMS methodology, we can selectively activate the three components and correlate the isotopic mass shift with precursor-dependent arrival time differences. This method enables assignment of the two main components as anomeric forms of the pentasaccharide, while the low-intensity component is assigned as an open-ring, intermediate from the mutarotation reaction occurring in solution. We then re-investigate three pentasaccharides as a mixture to explore applicability of this approach for multicomponent sample analyses. We therefore propose the use of high-resolution tandem IMS methodology to decipher glycosidic bond sequence without any derivatization.

## Methods

### Sample Preparation

Three pentasaccharides, 1,4-β-D-cellopentaose, maltopentaose, and α1–3,α1–3,α1–6 mannopentaose, were purchased from Dextra Laboratories Ltd. (Reading, UK). Water-^18^O was purchased from Sigma-Aldrich (Poole, UK). The pentasaccharides were dissolved in Water-^18^O to a concentration of 50 μM. Samples were incubated for 7 days in the dark to allow for oxygen exchange at the reducing end of the saccharide and diluted to 5 μM in 49.5:49.5:1 water, methanol, formic acid solution (WMA) just before MS analysis. Non-labeled samples were dissolved in H_2_O to a concentration of 50 μM and diluted further to 5 μM in WMA solution. The mixture of cellopentaose, maltopentaose, and branched mannopentaose was prepared in the ratio of 1.5:1.5:1 to account for different ionization efficiencies of the three saccharides.

### Mass Spectrometry

**Figure 1:**
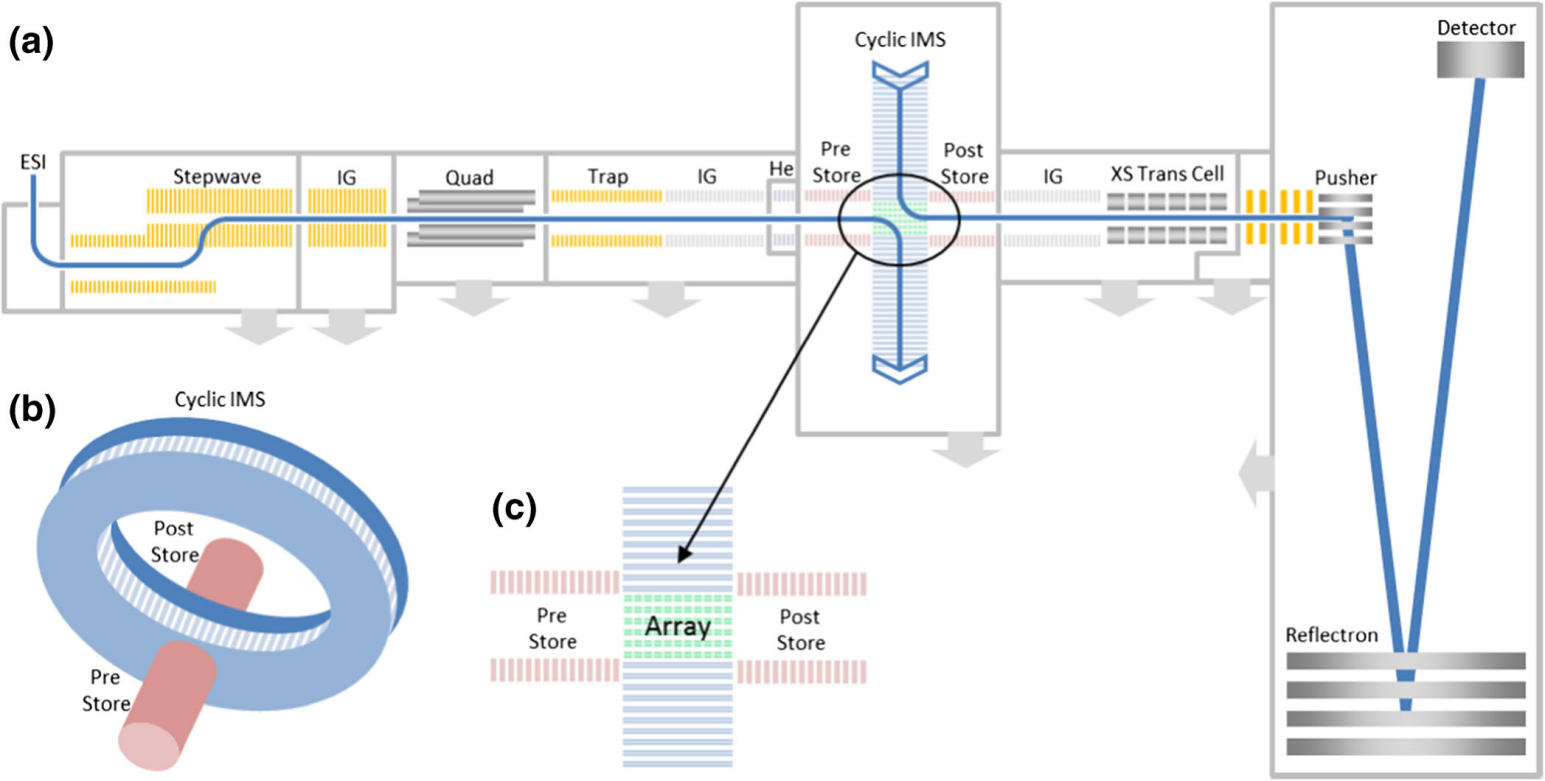
(**a**) Instrument schematic showing the Q-cIM-ToF geometry. (**b**) Cartoon showing the orthogonal arrangement of the cyclic IMS and neighboring ion optics. (**c**) Multi-function region

The pentasaccharide samples were analyzed in positive polarity and the species were ionized and detected as singly charged, sodium adducts [M + Na]^+^ (*m*/*z* 851). Samples were introduced into the instrument using a nano-ESI source (Waters Corp., Wilmslow, UK). The emitters (GlassTip™, New Objective, MA, USA) were held at 1.5–2 kV. The cIM instrument design is discussed in detail elsewhere (Giles et al. manuscript in preparation) and so will only be briefly covered here. The instrument schematic is presented in Figure 1. Ions are transferred from the source through the first vacuum stages using ion guide arrangements (StepWave), which propel ions towards the quadrupole mass filter. The subsequent trap cell allows storage and activation of mass-selected ions. The resulting ion packets are then transported into a helium cell and subsequent ion guide (pre-store) operating in nitrogen (2 mBar). Following this, there is a multi-function array (Figure 1c) of electrodes forming part of an orthogonal closed loop, the cIM separator (98-cm path length, single pass IM resolution ~ 65). The traveling wave (TW) direction in the array can be altered to either match those in the cIM device (i.e., during mobility separation) or to inject/eject ions from it. The resolution of the multi-pass separations scales as a square root of the number of passes: *R = A*(*nz*)^1/2^; where *A* is the single pass resolution, *n* is the number of passes, and *z* is the ion charge state. The single pass resolution is determined using compounds of known collision cross section values (in this case, the singly charged, reverse sequence peptide pair: GRGDS, SDGRG). The single pass resolution A is typically measured to be between 65 and 75 [28]. The control software GUI enables creation of custom sequences of functions to facilitate selective ejection of ions from the cIM device and/or activation followed by further separation of product ions. Two possible sequences utilizing IMS/IMS and IM isolation are illustrated in Figures S1 and S2. Post cIM, ions are transported through a second ion guide (post-store) and on to the oa-ToF via a segmented quadrupole (XS) transfer cell. The transfer cell allows fragmentation of the mobility-separated ions. The oa-ToF features an offset V geometry allowing *m*/*z* measurements at resolutions in excess of 60,000 FWHM. Instrument control and data acquisition is achieved via a flexible and customized web–based GUI. Data are processed using Masslynx (v4.1) and Driftscope (v2.9) (Waters Corp., Wilmslow, UK).

## Results and Discussion

### High-Resolution IM Separations of the Pentasaccharide Mixture

**Figure 2:**
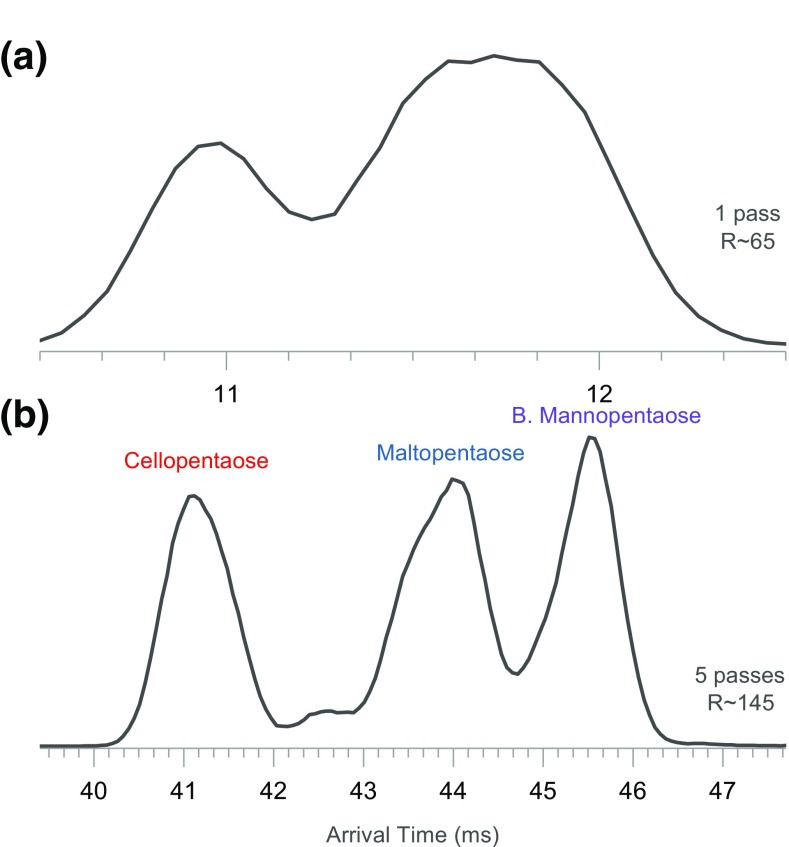
Ion mobility spectra of a mixture of cellopentaose, maltopentaose, and branched mannopentaose. (**a**) IM spectrum of the mixture after 1 pass and (**b**) after 5 passes around the cIM separator

Figure 2a–c shows the IM spectra of the mixture after 1 and 5 passes around the cIM separator. After 5 passes, the three pentasaccharides are resolved (Figure 2b). The arrival time distributions (ATD) are asymmetrical, likely due to presence of unresolved components. These features will be investigated in the latter sections of this report. The arrival time includes a so called “dead time”—the time ions spend traveling from the array to the detector. For the most mobile pentasaccharide (cellopentaose), the dead time is ~ 3.35 ms, whereas for the slowest (branched mannopentaose) it is ~ 3.44 ms.

Mobility-separated ions can be activated and dissociated in the transfer cell as shown in the 2D plot in Figure S3. A whole range of sodiated, mobility-separated product ions are observed; however, the arrival time dimension appears congested. We observe very different dissociation efficiencies for the three separated pentasaccharides as partly evidenced by the arrival time distributions (ATDs) of the remaining precursor ions at high collision energy (Figure S3, inset).

### MS/MS of ^18^O-Labeled Pentasaccharides

In order to facilitate the assignment of product ions, we investigated the three pentasaccharides which were isotopically labeled by heavy oxygen as described in Ropartz et al. [27]*.* A shift of two mass units resulting from incorporation of the ^18^O atom end allows the product ions which contain the reducing end (named X, Y, and Z [29]) to be identified. The products which do not contain the reducing end (called A, B, and C and consecutive fragments [29]) do not exhibit the mass shift. Example mass spectra of labeled and non-labeled precursors are presented in Figure S4. Initially, we activated the labeled and non-labeled pentasaccharide ions in the trap cell (before IM separation) and mass spectra were recorded, as shown in the Figure S5. Upon first inspection of the MS/MS data, it appeared that the maltopentaose and branched mannopentaose ions at 689 *m*/*z* are converted to 691 *m*/*z*, confirming that those species are almost exclusively Y-type ions (i.e., contain the reducing end of the saccharide, Figure S6). No shift was observed for the 671 *m*/*z* ions of maltopentaose, suggesting they are almost exclusively B-type. A parallel effect is seen for species at 527 and 509 *m*/*z*. On the contrary, for cellopentaose, a mix of 689 and 691 *m*/*z* products is observed, suggesting presence of both Y- and C-type ions. Interestingly, activation of cellopentaose yields a significant amount of ions at 833 *m*/*z*. This product ion does not contain the label; however it must originate from the isotopically labeled precursor (Figure S5a and b). Consequently, the 833 *m*/*z* ions are assigned as the beta elimination of H_2_^18^O from the reducing end.

### IMS/IMS of Pentasaccharide Conformers

**Figure 3:**
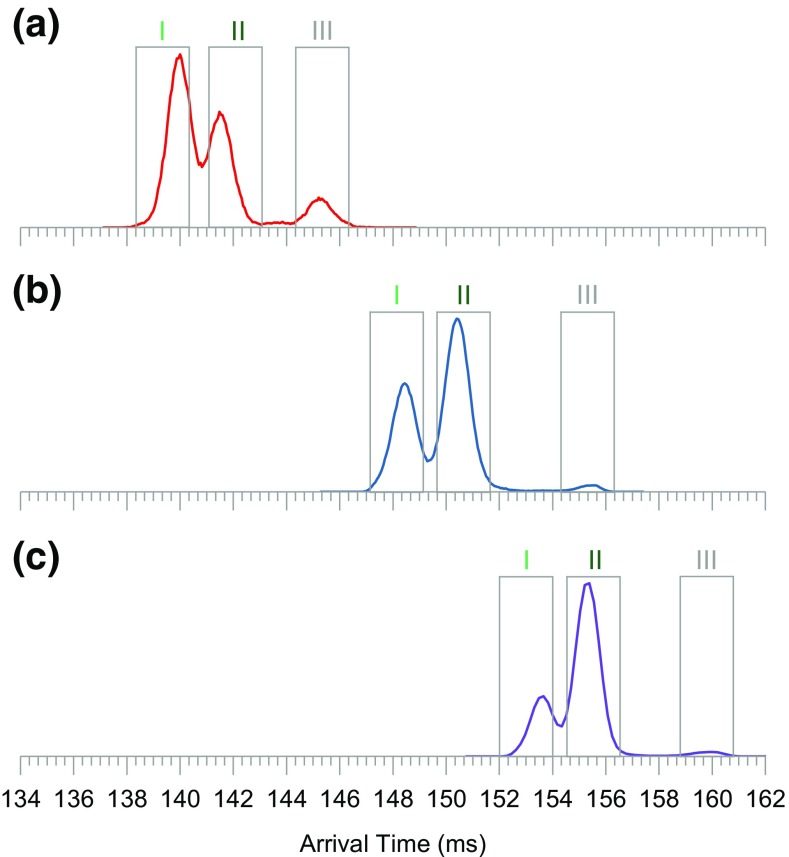
Ion mobility spectra of the three, isotopically labeled pentasaccharides after 18 passes around the cIM device: (**a**) cellopentaose, (**b**) maltopentaose, (**c**) branched mannopentaose. Each compound appears to consist of three components. Gray rectangles correspond to IM selection regions used in the IMS/IMS experiment

Figure 3a–c shows the IM spectra of the three, ^18^O-labeled pentasaccharides, infused separately, after 18 passes around the cIM separator (calculated IM resolution of ~ 275). The mobility spectrum of each pentasaccharide reveals two main components (I and II) and a third, low-intensity species (III). Interestingly, the relative intensities of the resolved components differ between the three compounds. The resolved components of each precursor can be investigated in detail using the IMS/IMS methodology (Figure S1). A control sequence was set up where precursors I, II, and III were selectively ejected (Figure 3a–c, gray boxes) to the pre-store and then re-injected to the cIM array with increased collision energy to generate product ions. The product ions were then subjected to a single pass IM separation followed by *m*/*z* measurement.

**Figure 4:**
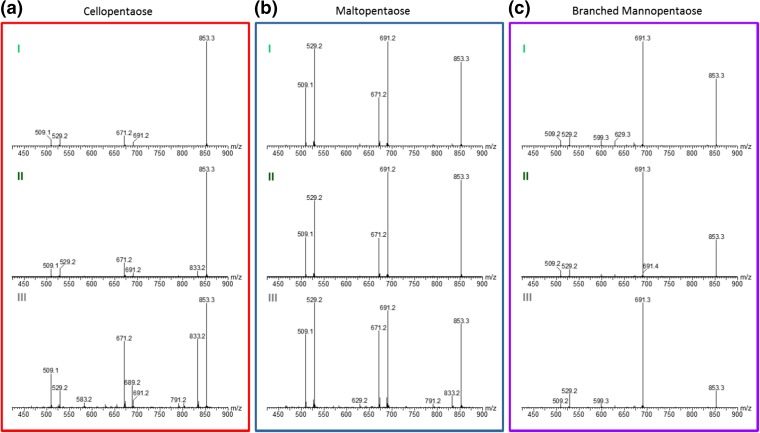
Mass spectra obtained after IMS/IMS experiments for three, ^18^O-labeled pentasaccharides. (**a**) Mass spectra of three IM separated components of cellopentaose. (**b**) and (**c**) Corresponding spectra of maltopentaose and branched mannopentaose respectively. Panel colors correspond to traces in Figure 3

The resultant mass spectra are presented in Figure 4. It is apparent that for cellopentaose, component III yields the highest number of product ions. Figure 4 a shows fragmentation spectra of components I, II, and III, where we can see that ions at 691 *m*/*z* (i.e., containing the reducing end) are produced from the two main precursors, while a large yield of the 689 *m*/*z* fragment (i.e., not containing ^18^O) is produced from component III.

For the cellopentaose and maltopentaose, a significant yield of ions at 833 *m*/*z* (H_2_^18^O loss) is produced from precursor III. These species have been already assigned as reducing end cleavage. It is however interesting that these ions are produced predominately from component III. Lack of the isotopic shift and an increased abundance of other intracyclic cleavage products (791 *m*/*z*, 629 *m*/*z*, and 583 *m*/*z*) suggest that component III corresponds to the precursor with an open-ring conformation of the reducing end. Continuing with this reasoning, we can postulate that the 691 *m*/*z* product of cellopentaose component III (Figure 4a III) is a Y-type ion, i.e., containing the reducing end *but* in the open-ring form. Interestingly, the C-type ions at 689 *m*/*z* appear to be created exclusively from the open-ring precursor. Unlike maltopentaose and cellopentaose, the mass spectra of products originating from the I, II, and III components of branched mannopentaose appear very similar (Figure 4c). This can be rationalized by the presence of 1–3 and 1–6 branches, which was shown previously to strongly interfere with cross-linked cleavages [30].

**Figure 5:**
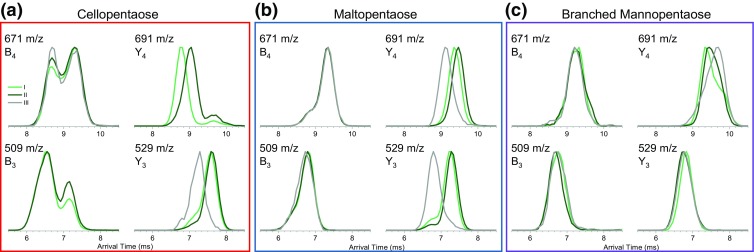
IMS/IMS spectra of selected product ions of (**a**) cellopentaose, (**b**) maltopentaose, and (**c**) branched mannopentaose. Panel colors correspond to traces in Figure 3. Product ions originating from components I, II, and III are represented by light green, dark green, and gray traces, respectively. The 509 and 691 *m*/*z* product data for cellopentaose from component III (low level) and IMS/IMS spectra of other product ions and the remaining precursor ions are presented in Figures S7–S9

Product ions of each precursor component can be investigated further using a second stage of IM separation (IMS/IMS) and the data are shown in Figure 5. Strikingly, the IM spectra of the cellopentaose and maltopentaose product ions contain features which appear correlated with the precursor component while some others exhibit no difference. For example, ions at 509 *m*/*z* and 671 *m*/*z* originating from precursors I and II appear to have very similar ATDs (Figure 5a, b, light and dark green traces in 509 and 671 *m*/*z* plots). On the other hand, ions at 529 *m*/*z* and 691 *m*/*z* originating from the faster precursor (I) are more mobile than the same *m*/*z* ions produced from the slower precursor (II). In the case of the 691 *m*/*z* products of cellopentaose, this effect is most obvious.

In several cases, the ATDs exhibit a bimodal distribution. Neither of the peaks in the bimodal distribution of the 671 *m*/*z* ions of cellopentaose appears correlated with the precursor component; however, some differences in abundance are observed. On the other hand, the 691 *m*/*z* products of cellopentaose also exhibit a bimodal distribution (smaller component at ~ 9.7 ms), where one component does exhibit the precursor-dependent difference while the other does not. Somewhat similar effects are seen for the branched mannopentaose products. The shape of the ATD of *m*/*z* 691 of branched mannopentaose indicates the presence of multiple conformers, these are likely correlated with different mechanisms leading to the loss of a lateral branching (Figure S6). In this case, the single pass IM separation in the second stage appears insufficient to resolve the overlapping components. These species were subjected to three passes around the cIM and indeed, several distinct conformers are resolved (Figure S11). Such species can be investigated comprehensively using IMS^n^ and this will be addressed in future work.

**Figure 6:**
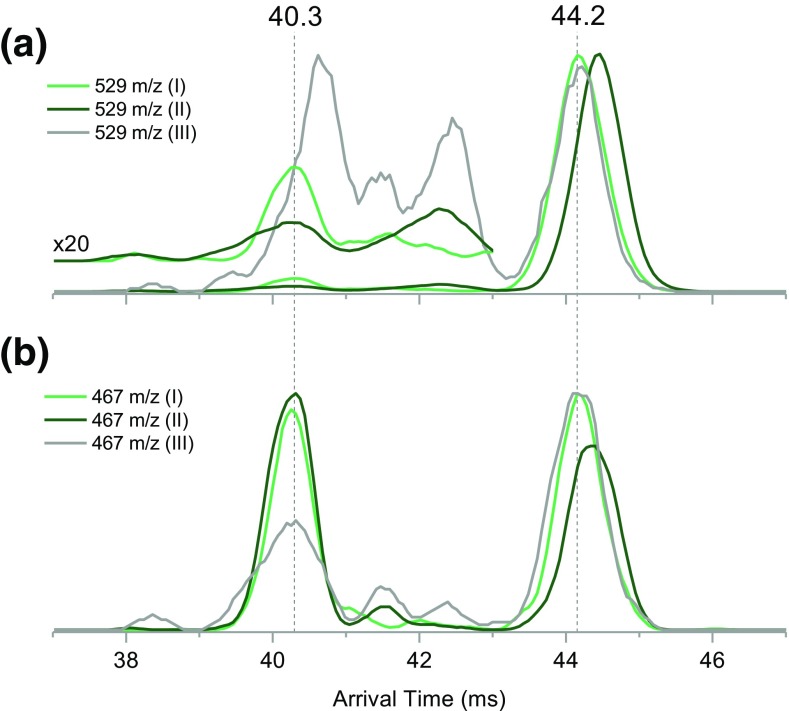
(**a**) Arrival time distributions of 529 *m*/*z* ions (Y_3_) originating from maltopentaose precursors I, II, and III after six passes around the cIM. (**b**) Arrival time distributions of 467 *m*/*z* ions (^0,2^A_3_) produced by activating IM separated Y_3_ ions in the post-mobility transfer cell of the instrument

From the data presented in Figures 4 and 5, it is therefore clear that components I and II in the pentasaccharide precursor ATD are due to the structural differences at the reducing end, which is likely to be due to the anomeric conformation (Figure S13). The remaining feature (III) is likely to be an open-ring conformation of the reducing end. It is intriguing that some of the product ions exhibit bimodal distributions where only one feature appears correlated with the precursor component. In the case of the maltopentaose 529 *m*/*z* ions, the non-correlated feature appears very similar in arrival time to the 529 *m*/*z* ions originating from open-ring conformer III (both at 6.7 ms). This hints that the “non-correlated” features in the IM spectra of ions containing the ^18^O label could correspond to open-ring products originating from closed-ring precursors. To test this hypothesis, we investigated the yield of the ^0,2^A_3_ intracyclic cleavage products (loss of 62 Da from ^18^O-labeled Y_3_ ions) produced from the “non-correlated” features. The 529 *m*/*z* fragment ions (^18^O-labeled Y_3_) produced from maltopentaose-I, maltopentaose-II, and maltopentaose-III were subjected to higher resolution IM separation (6 passes) *and* additional post-IM fragmentation in the transfer cell (Figure 1, “XS trans cell”). Because no IM separation takes place after activation in the transfer cell, the ^0,2^A_3_ products (467 *m*/*z*) will have arrival times aligned with the features of the Y_3_ ATDs they originated from (Figure 6).

Higher resolution separation reveals several features in the previously unresolved leading edge of the 529 *m*/*z* ions originating from maltopentaose-I and maltopentaose-II (Figure 5b, 529 *m*/*z*, light and dark green traces). In fact, the leading edge consists of at least two low-intensity components (Figure 6a, × 20 inset). The fastest of those components (40.3 ms) is “non-correlated” and produces the highest yield of the 467 *m*/*z* ions (Figure 6b, 40.3 ms). This leads us to postulate that the 529 *m*/*z* species arriving at 40.3 ms correspond to open-ring Y_3_ fragments originating from closed-ring maltopentaose precursors, i.e., isomerization during activation. This hypothesis is in line with the retro-aldol mechanism proposed by Bythell et al. [31] (Figure S10). We now focus our attention on the Y_3_ ions originating from the open-ring maltopentaose-III (Figure 6a, gray trace). Higher resolution data reveals that the ATD of this product consist of at least four components. Features with arrival times between 39 and 43 ms produce varying yields of the 467 *m*/*z* ions. The highest yield of 467 *m*/*z* looks to be produced from the low-intensity feature at 40.3 ms, which appears unresolved in the ATD of Y_3_ precursor (Figure 6a, gray trace). Intriguingly, the slowest component of the Y_3_ ions produced from maltopentaose-III is very well aligned with ATD of 529 *m*/*z* product of maltopentaose-I (Figure 6a, 44.2 ms, gray and light green traces). This suggests another gas-phase isomerization reaction, in which an open-ring Y_3_ ion is converted into *only* one of the closed-ring forms. We note that the latter conclusion is rather preliminary and will require confirmation in future work. If this hypothesis proves correct, it could form a basis for assignment of α anomers and β anomers.

### IMS/IMS of the Non-labeled Pentasaccharides Mixture

**Figure 7:**
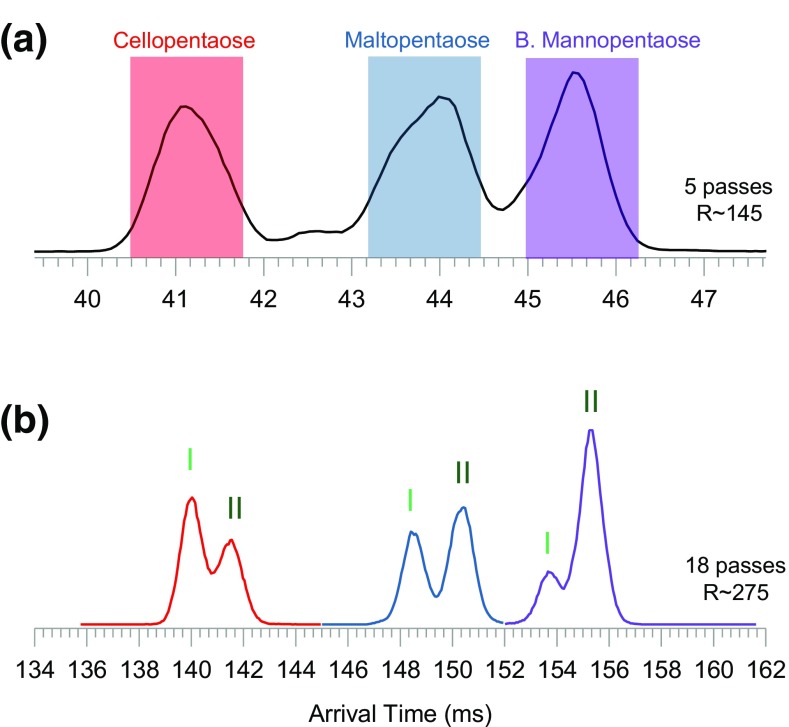
(**a**) Ion mobility spectra of a mixture of the three non-labeled pentasaccharides after 5 passes. After 5 passes, the main components of each compound (denoted by the colored boxes) are isolated in the cIM device (Scheme detailed in Figure S2) and subjected to a further 13 passes. (**b**) IM spectra of isolated species after 18 passes in total (5 + 13); components I and II are now resolved for each saccharide

In order to benchmark the robustness of the IMS/IMS methodology for saccharide characterization, we re-investigated the three non-labeled pentasaccharides as a mixture. Figure 7 a shows the IM spectrum of the mixture after 5 passes around the cIM separator. After 5 passes, the three pentasaccharides are resolved. As expected, the ATD peaks are asymmetrical due to presence of unresolved components. In the cIM, an effect known as “wrap around” (Giles et al. manuscript in preparation) can occur where the fastest ions start to overtake the slowest ones. For the mixture of three pentasaccharides, “wrap around” would occur after the 5th pass. Using the multi-function capability of the instrument control, one can isolate species of interest in the cIM device while the rest of the ions are ejected (Figure S2). Such an approach is used here to obtain the higher resolution IM spectra for the three compounds. Colored boxes in Figure 7 a depict ion populations which are sequentially isolated in the cIM device. This is done separately for each mobility selection window. After subsequent 13 passes (18 passes in total, Figure 7b), all three pentasaccharides appear resolved into two (I and II) components. The trade-off of this approach is that if separation at the point of isolation (i.e., after 5 passes) is not sufficient to resolve all components (i.e., III), they will also be ejected out and therefore not be present in latter spectra. This is why component III is missing in Figure 7 b.

**Figure 8:**
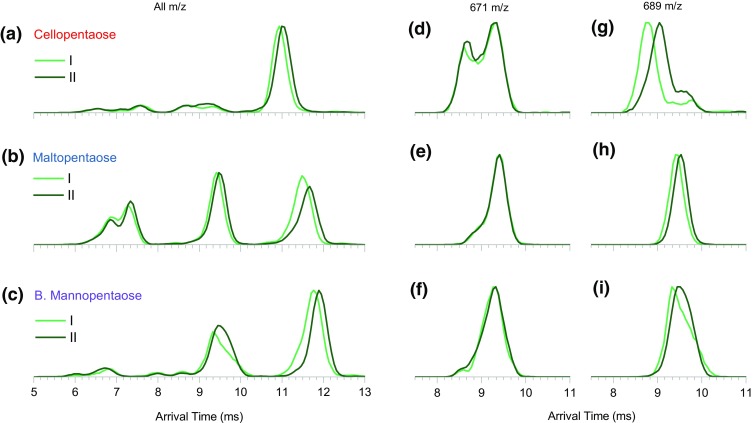
(**a**)–(**c**) Full *m*/*z* range IM spectra of activated I (light green) and II (dark green) components of the three non-labeled pentasaccharides analyzed as a mixture. (**d**)–(**f**) IM spectra of fragments at 671 *m*/*z*, showing no precursor-dependent arrival time difference. (**g**)–(**i**) IM spectra of fragments at 689 *m*/*z* which exhibit precursor-dependent arrival time difference

Components I and II of each precursor can be now be re-investigated by the IMS/IMS methodology. An experiment was set up where faster and slower precursors were ejected to the pre-store and then re-injected with increased collision energy to generate product ions. These ions were subjected to single pass IM separation and *m*/*z* measurement. The resultant mass spectra are presented in the Figure S12 and in general, they do not show precursor-dependent differences. The combined IM spectra of the activation products are presented in Figure 8a–c. Peaks between 10.5 and 12.5 ms correspond to the remaining precursor, which compares with corresponding data in Figure 2 a. Peaks between 6 and 10.5 ms correspond to product ions. From the combined (all *m*/*z*) tandem IM spectra (Figure 8a–c), it is apparent that the three pentasaccharides exhibit different fragmentation patterns. Again, the IM spectra of products of each pentasaccharide contain features which appear correlated with precursor component while some others exhibit no difference. For example, ions at 671 *m*/*z* appear to have superimposable ATDs, no matter which precursor component they originated from (Figure 8d–f). On the other hand, ions at 689 *m*/*z* originating from the faster precursor are more mobile than the same *m*/*z* ions produced from the slower precursor. The 689 *m*/*z* products of cellopentaose exhibit a bimodal distribution, where one component does exhibit the precursor-dependent difference while the other does not (Figure 8g). Overall, the observed effects are consistent with those described in the previous section. In the case of the products of branched mannopentaose-II, the analysis is somewhat hindered by the presence of the overlapping maltopentaose-III precursor (Figure 3b, c).

## Conclusions

Using the Q-cIM-ToF instrument, we have demonstrated the utility of high-resolution IM separation and IMS/IMS functionality, which can be extended to IMS^n^-type experiments. The tandem IMS approach proved extremely flexible and valuable in unraveling new information regarding the fragmentation of oligosaccharides. With the aid of isotopic labelling, we were able to correlate the assignments of features resolved in IMS/IMS-MS experiments. We show that the performance of the instrument is sufficient to resolve three components of three isomeric pentasaccharides. We cannot exclude the possibility that the coordination of sodium cation is different between conformers I, II, and III. However, the data implies that the difference would have to be *induced* by the structure of the reducing end (i.e., anomerism). There are two main experimental findings supporting this conclusion: (1) the structural difference is preserved only in the Y-type product ions, as verified by the IMS/IMS and ^18^O-labelling approaches; (2) If sodium coordination was indeed the source of a difference between components I and II, we would expect to see “precursor correlated” B-type ions. Although, in some cases multiple conformers of B-type ions are observed, they are never correlated with precursor structure. Therefore, we assign components I and II as anomers. Component III is assigned as an open-ring form—we base this conclusion on the high yield of the intracyclic cleavage fragments produced from this component. As an additional control experiment (suggested by one of the reviewers), we performed high-resolution IM separation of a sodiated, non-reducing oligosaccharide (melezitose, Figure S14). As expected, the ATD presents as a single feature, in line with the fact that there is no possibility of a ring-opening reaction in solution. For completeness of the above work, a definitive assignment of components I and II as α and β anomers would be necessary. This could be achieved using spectroscopy, as demonstrated by several groups [32–34].

In the course of this work, we observed differences in abundance of different anomers; we initially assumed that these were due to the differences in sodiation efficiency of the anomeric forms. However, the abundance ratios observed previously by Deng et al. [23] are in some cases different from the ones presented here and further work would be needed to explore origins of this effect. In addition to the three isomeric forms of the precursors (I, II, and III), on several occasions, we identified multiple structures of product ions, some at low abundance. We tentatively assign these as the open-ring products originating from the closed-ring precursors and, in the case of branched mannopentaose, as isomers resulting from different losses of lateral branching. In addition, some of the data (i.e., Figure 6) suggest that activation of open-ring Y-type ions can yield species with mobility very similar to those originating from closed-ring Y-type ions. It remains to be investigated whether these data are evidence of the ring-closing reaction occurring in the gas phase.

We explored the applicability of the IMS/IMS technique (without ^18^O-labelling) for the analysis of the mixture of the three compounds. This experiment utilized the additional IM isolation capability of the instrument. Following this step, anomeric components of each pentasaccharide were separated at high resolution and analyzed using IMS/IMS. The low-intensity component III was not fully resolved in all cases, which highlights the limitation of this approach. This issue would be more pronounced when concentrations and/or ionization efficiencies of a mixture of components were significantly different. A parallel shortcoming is commonly encountered in MS/MS workflows, where precursor ions have the same or very similar *m*/*z* ratios.

It is yet to be established whether *all* reducing carbohydrates are resolvable into three (or more [26]) components. If so, a very specific carbohydrate identification workflow could be based on the combination of the *m*/*z* value of the precursor and product ion, their CCS values, and their relative abundance. In addition to such “fingerprint”-based identification, we can postulate that IMS^n^-MS experiments would allow structural elucidation of large carbohydrates with subtle modifications such as the nature of the branching pattern including the linkage anomerism. Importantly, IMS/IMS-MS methodology was shown to be useful in deciphering conformers of the reducing end (anomers and open ring). Thus, it should also allow discrimination of the above species from multiple conformations of the *same* saccharide species arising from different metal cation binding sites. As already stated, such experiments are possible with this Q-cIM-MS instrument geometry and will be used to explore these interesting compounds further.

## Electronic Supplementary Material

ESM 1 (DOCX 1.06 mb)
