# Supplementary material for: Cyclic Ion Mobility Mass Spectrometry Distinguishes Anomers and Open-Ring Forms of Pentasaccharides
Source: J Am Soc Mass Spectrom. 2019 Apr 11;30(6):1028–37. doi: 10.1007/s13361-019-02168-9 (PMC6517361; doi:10.1007/s13361-019-02168-9)
Supplement: Supplementary file 1 — (DOCX 1.06 mb) [file 13361_2019_2168_MOESM1_ESM.docx]

**Supporting Information**

**Cyclic Ion Mobility Mass Spectrometry distinguishes Anomers and Open-chain forms of Pentasaccharides**

Jakub Ujma^1^, David Ropartz^2^, Kevin Giles^1^, Keith Richardson^1^, David Langridge^1^, Jason Wildgoose^1^, Martin Green^1^, Steve Pringle^1^.

^1^ Waters Corporation, MS Research, Wilmslow, UK.

^2^ INRA, UR1268 Biopolymers Interactions Assemblies, Rue de la Géraudière B.P. 71627, F-44316 Nantes, France.

**
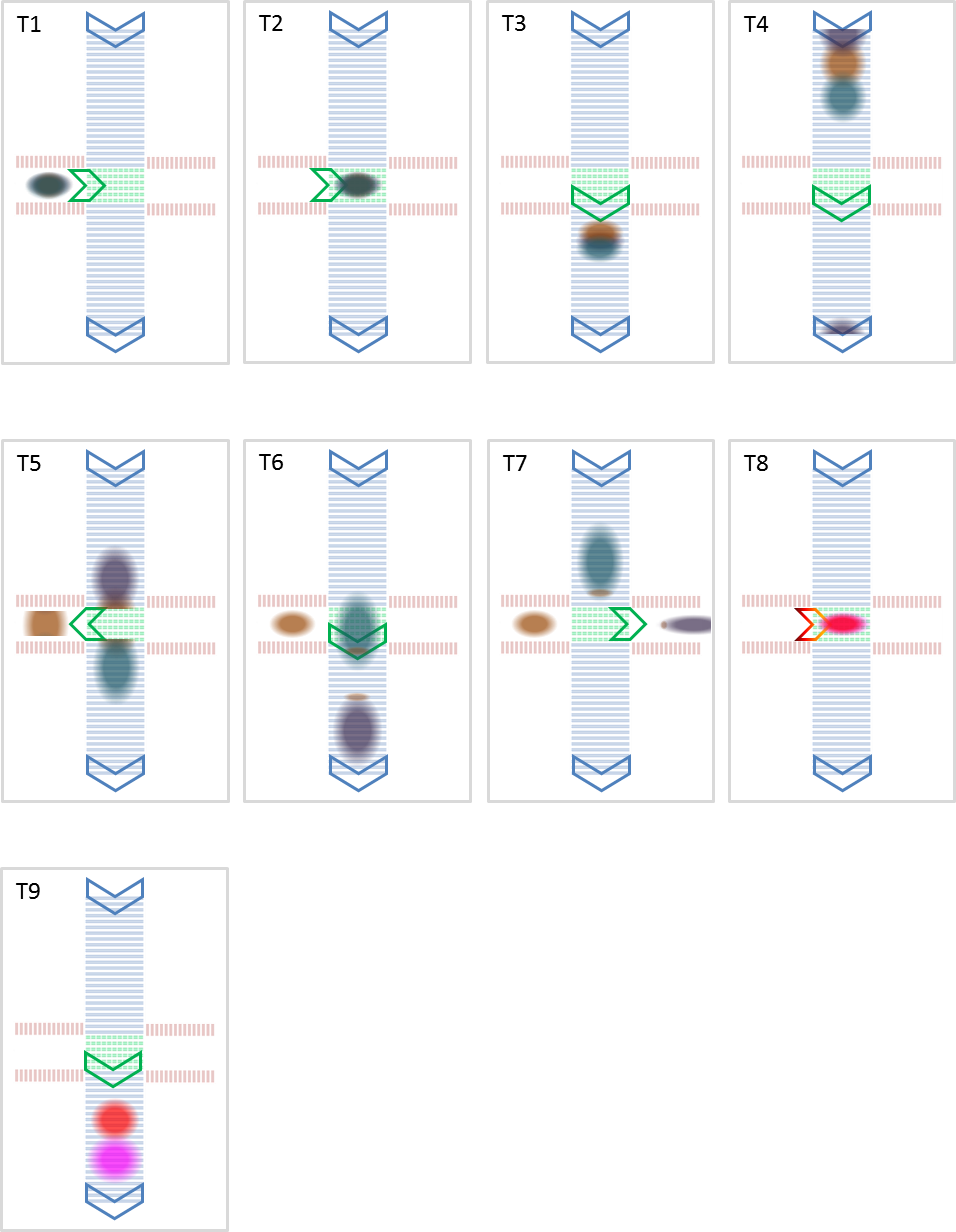
**

Figure S1. Events in IMS/IMS mode of operation. Time points 1 and 2 (T1 and T2): ions are injected from store (red) into the array (green). T3: direction of T-waves in the array (green arrow) changes to match those in cIM region (blue arrows); ions begin to drift around cIM device. T4: after certain number of passes three species appear separated. T5: IM selection; as brown population of ions drifts through the array region, the array T-wave direction changes and the selected ion population is ejected into the store. T6, T7: ions remaining in the cIM travel for an additional pass around cIM before being ejected out. T8: ions from the store are re-injected into the array at high energy and form product ions. T9: Product ions originating from brown precursor population are mobility separated after certain number of passes.

**
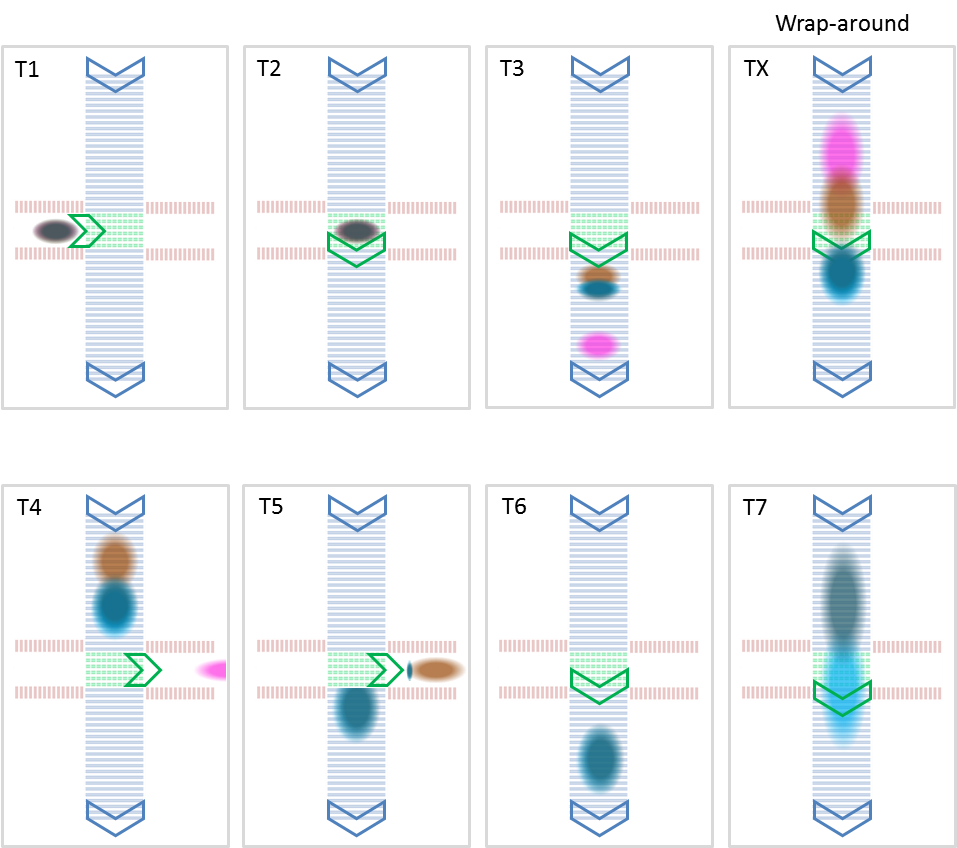
**

Figure S2. Events in IM isolation mode of operation. Time points 1 and 2 (T1 and T2): ions are injected from store (red) into the array (green). T3: direction of T-waves in the array (green arrow) changes to match those in cIM region (blue arrows); ions begin to drift around cIM device. TX: Wrap-around limit - after certain numbers of passes, the most mobile ions (pink) begin to overtake the least mobile (brown) ions. T4: time point before wrap-around limit is approached. The most mobile ions are selectively ejected out. T5: blue ions are allowed to proceed to subsequent pass, the brown ion population is ejected out. T6: blue ion population is isolated in the cIM device. T7: after certain number of passes, blue ion population appear resolved into two components.


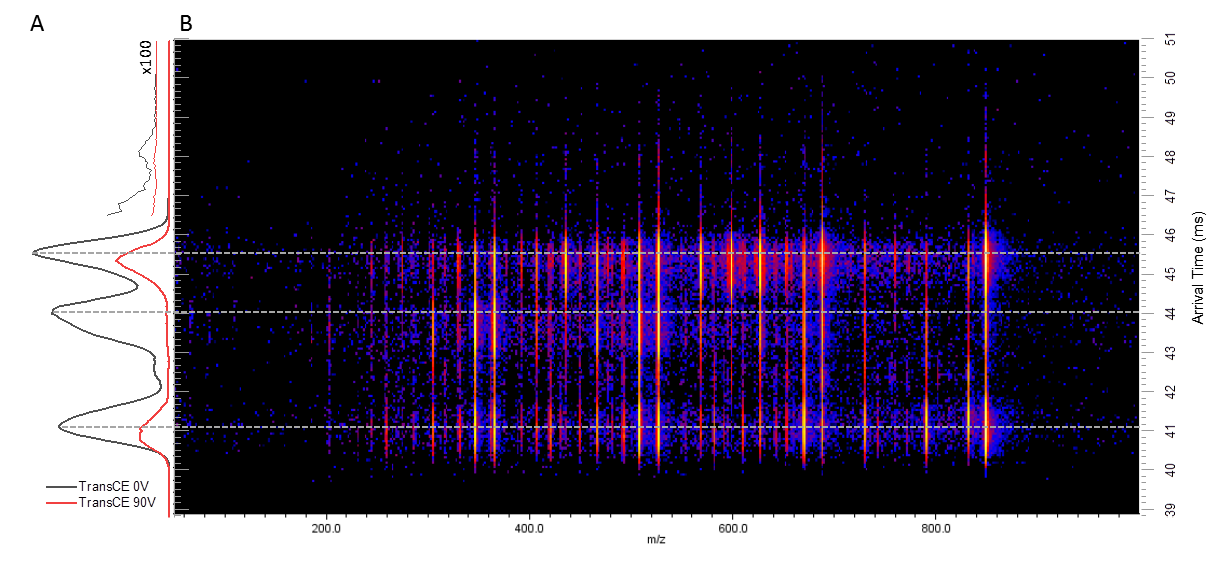


Figure S3. 2D plot of mobility separated activation products of 851 m/z ions. Inset (left): the ATDs of 851 m/z ions at transfer collision energy voltage settings of 0V and 90V.

Figure S4. Pentasaccharide labelling and mass selection – Branched Mannopentaose. (a) isotopic distribution of sodiated of branched mannopentaose. (b) Quadrupole selected 851.3 m/z ion. (c) isotopic distribution of branched mannopentaose after incubation for 7 days in water-^18^O. (d) Quadrupole selected 853.3 m/z ion corresponding to ^18^O-labelled branched mannopentaose.


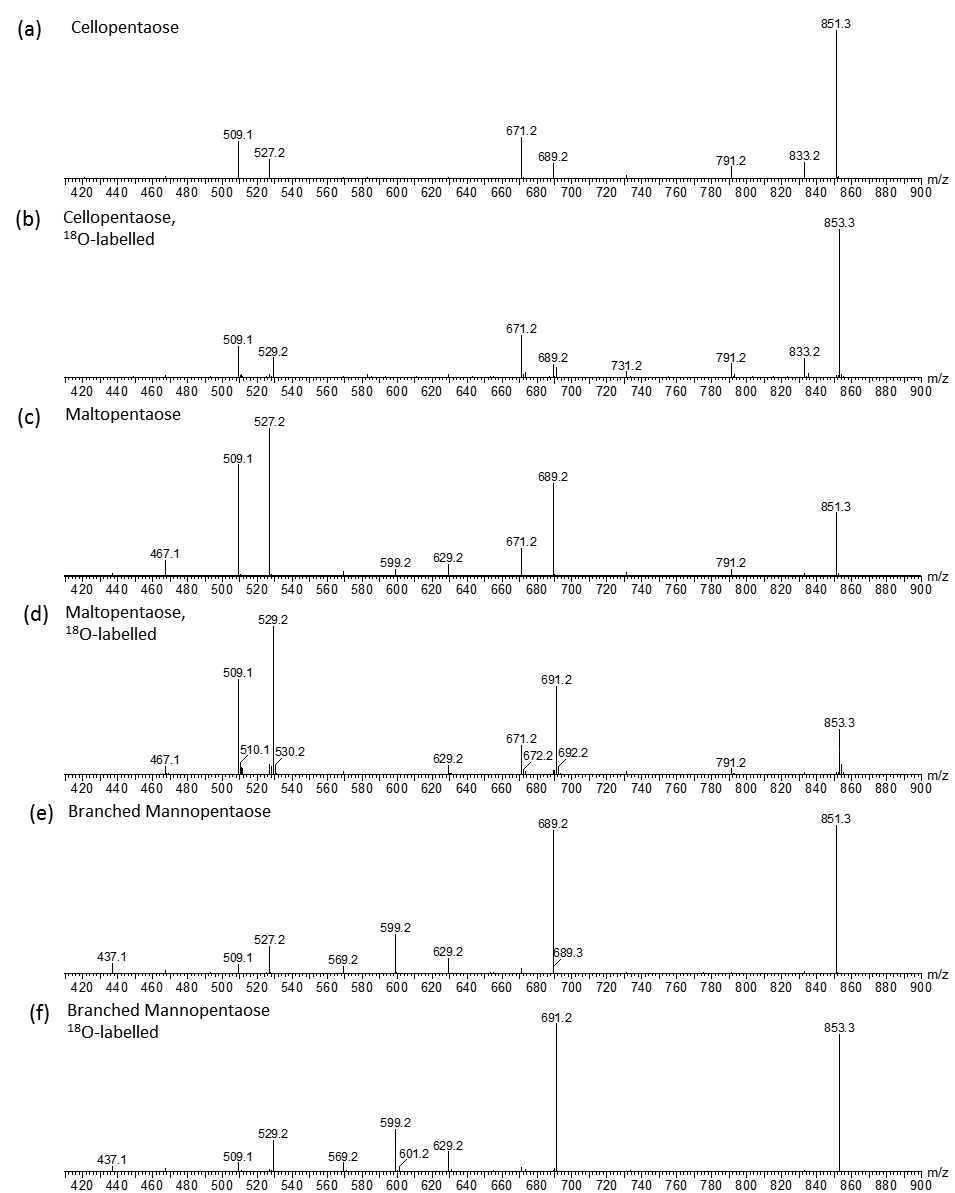


Figure S5. MS/MS spectra of non-labelled pentasaccharides (a, c, e) and their ^18^O-labelled counterparts (b, d, f). Activation was performed in the trap at 90V collision energy.

*
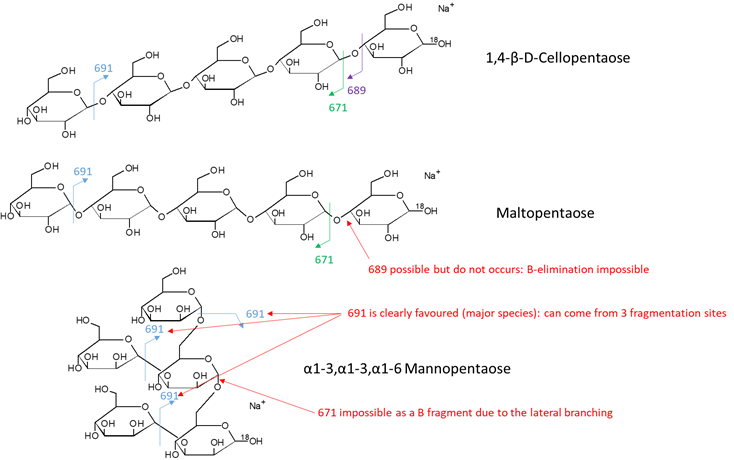
*

Figure S6. Scheme showing origin of product ions of three pentasaccharides.

Figure S7. IMS/IMS spectra of cellopentaose fragments originating from three components: I (light green traces), II (dark green traces) and III (grey traces).

Figure S8. IMS/IMS spectra of maltopentaose fragments originating from three components: I (light green traces), II (dark green traces) and III (grey traces).

Figure S9. IMS/IMS spectra of branched mannopentaose fragments originating from three components: I (light green traces), II (dark green traces) and III (grey traces).

**
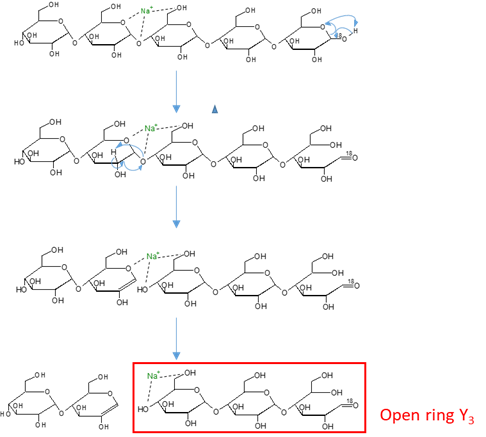
**

Figure S10. Proposed mechanism of formation of "open-ring" Y_3_ product ions of maltopentaose based on work of Bythell *et al.* in Cationized Carbohydrate Gas-Phase Fragmentation Chemistry. J. Am. Soc. Mass Spectrom. 28, 688-703 (2017).

Figure S11. 691 m/z products of branched mannopentaose after three passes around the cIM device. Higher resolution (R~110) data shows presence of multiple components.


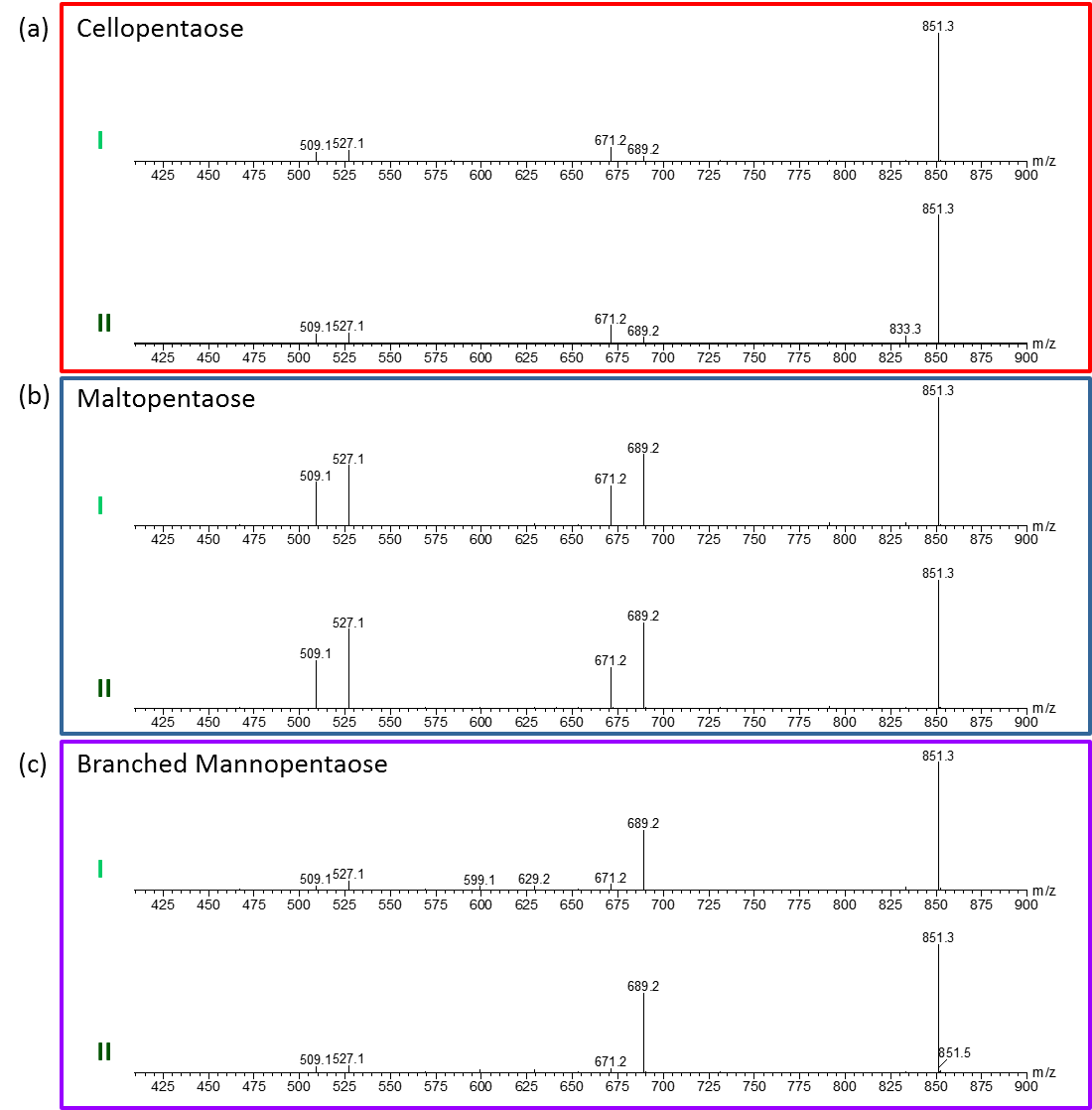


Figure S12. MS spectra of product ions generated in IMS/IMS experiment where three pentasaccharides are infused as a mixture.


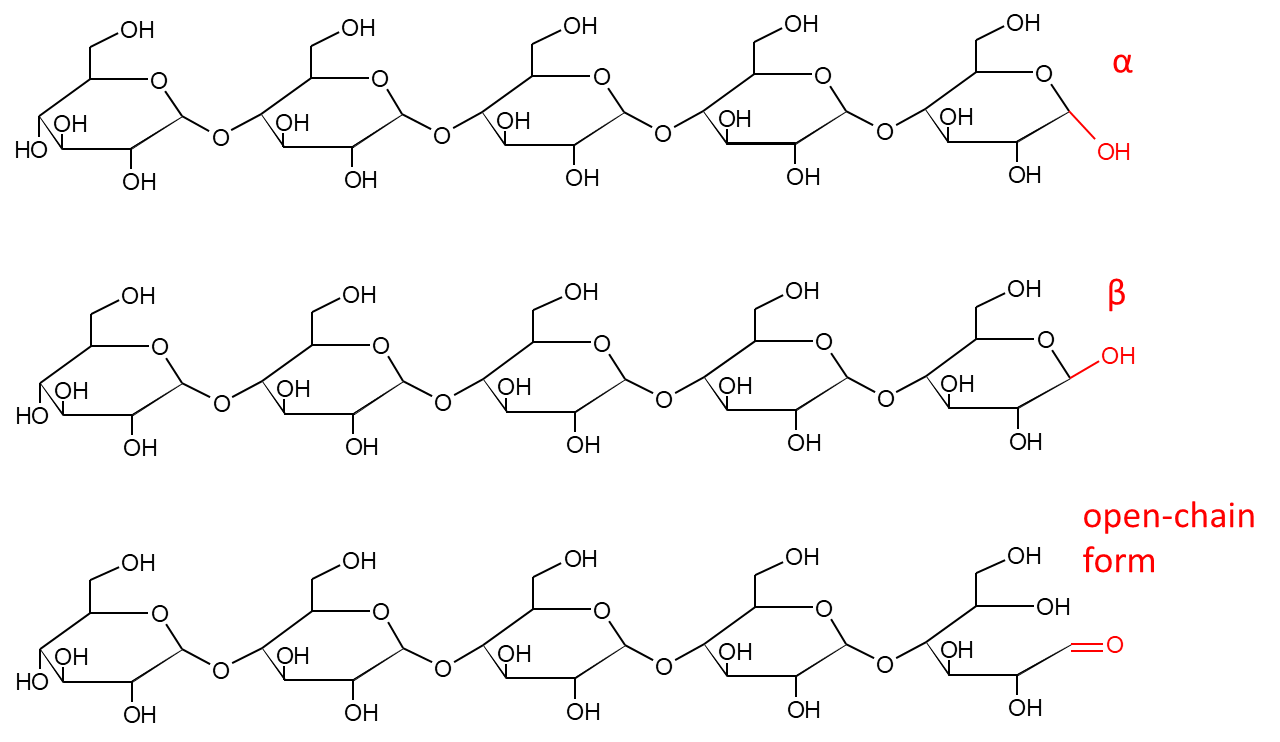


Figure S13. Alpha, beta and open chain forms of maltopentaose.

Figure S14. Ion mobility data for [Melezitose+Na]^+^ ions after 5 and 18 passes around the cyclic IM cell.
